# Facilitators and barriers of HPV vaccination: a qualitative study in rural Georgia

**DOI:** 10.1186/s12885-024-12351-1

**Published:** 2024-05-15

**Authors:** Courtney N. Petagna, Stephen Perez, Erica Hsu, Brenda M. Greene, Ionie Banner, Robert A. Bednarczyk, Cam Escoffery

**Affiliations:** 1 Department of Behavioral, Social, and Health Education Sciences, Rollins School of Public Health, Emory University, Atlanta, GA 30322 USA; 2 Southwest Health District, 8-2, Division of Public Health, Georgia Department of Public Health, Albany, GA 31710 USA; 3 Hubert Department of Global Health, Rollins School of Public Health, Emory University, Atlanta, GA 30322 USA

**Keywords:** Adolescent, Parents, Young adults, Providers, Health systems, HPV vaccination, Cancer prevention, Rural health

## Abstract

**Introduction:**

Human papillomavirus (HPV) vaccination protects against HPV-associated cancers and genital warts. Healthy People 2030 goal for HPV vaccine uptake is 80%, but as of 2021, only 58.5% of adolescents are up to date in Georgia. The purpose of the study is to assess the attitudes, vaccine practices, facilitators, and barriers to receiving the HPV vaccine in southwest Georgia.

**Methods:**

We conducted 40 semi-structured interviews in the United States from May 2020-Feburary 2022 with three different audiences (young adults, parents, and providers and public health professionals) guided by the P3 (patient-, provider-, practice-levels) Model. The audiences were recruited by multiple methods including fliers, a community advisory board, Facebook ads, phone calls or emails to schools and health systems, and snowball sampling. Young adults and parents were interviewed to assess their perceived benefits, barriers, and susceptibility of the HPV vaccine. Providers and public health professionals were interviewed about facilitators and barriers of patients receiving the HPV vaccine in their communities. We used deductive coding approach using a structured codebook, two coders, analyses in MAXQDA, and matrices.

**Results:**

Out of the 40 interviews: 10 young adults, 20 parents, and 10 providers and public health professionals were interviewed. Emerging facilitator themes to increase the uptake of the HPV vaccine included existing knowledge (patient level) and community outreach, providers’ approach to the HPV vaccine recommendations and use of educational materials in addition to counseling parents or young adults (provider level) and immunization reminders (practice level). Barrier themes were lack of knowledge around HPV and the HPV vaccine (patient level), need for strong provider recommendation and discussing the vaccine with patients (provider level), and limited patient reminders and health education information around HPV vaccination (practice level). Related to socio-ecology, the lack of transportation and culture of limited discussion about vaccination in rural communities and the lack of policies facilitating the uptake of the HPV vaccine (e.g., school mandates) were described as challenges.

**Conclusion:**

These interviews revealed key themes around education, knowledge, importance of immunization reminders, and approaches to increasing the HPV vaccination in rural Georgia. This data can inform future interventions across all levels (patient, provider, practice, policy, etc.) to increase HPV vaccination rates in rural communities.

**Supplementary Information:**

The online version contains supplementary material available at 10.1186/s12885-024-12351-1.

## Introduction

Human papillomavirus (HPV) is a common sexually transmitted infection (STI) in the United States (US) with an estimated prevalence of 42.5 million people and an incidence of 13 million people per year [1]. HPV-associated cancers, including vulvar, vaginal, cervical, penile, anal, and oropharyngeal cancers, can develop years or decades following persistent HPV infection [2, 3]. Between 2015 and 2019, it was estimated HPV caused 47,199 new cancer cases each year [2]. Georgia has an incidence rate of 12.9 per 100,000 persons of all HPV associated cancers compared to the United States at 11.8 per 100,000 persons [4]. Additionally, Georgia is ranked in the top 15 nationally for having high cervical cancer incidence rates (7.4 per 100,000 persons) and the national incident rate is 6.5 per 100,000 persons [4]. Due to the high incidence rates of HPV associated cancers, the Georgia Cancer Plan: 2019–2024 made targeting HPV associated cancers a priority in an effort to support cancer prevention efforts [5]. The objective related to this priority is (Objective 1): “To increase the number of females and males who complete the HPV vaccine series in accordance with the Advisory Committee on Immunization Practices (ACIP) and recommendations” [5].

HPV vaccine was developed to prevent HPV associated cancers and genital warts; [6] currently HPV vaccine is one of two cancer prevention vaccines available globally [7]. Previous research determined each HPV vaccine is safe and has at least 96% efficacy for preventing HPV-associated cancers [8]. HPV vaccination was recommended in the US for adolescent females in 2006, and for adolescent males in 2011 [9]. The Advisory Committee on Immunization Practices (ACIP) recommends vaccination from as young as 9 years old to age 26, with the possibility of receiving the vaccine up to the age of 45 through a shared decision making process between the provider and the patient [10]. The earlier a person receives the HPV vaccine before engaging in sexual activity, the better protected they will be from HPV-associated cancers and genital warts [11]. If the vaccine is initiated prior to the 15th birthday, vaccine recipients need to complete a two-dose vaccine series; if the first dose is given after the 15th birthday, vaccine recipients need to complete a three-dose series [12]. Healthy People 2030 offers standardized 10-year measurable health objectives for the United States. Among their target goals is to have 80% of adolescents aged 13 to 15 receive all recommended doses of the HPV vaccine. As of 2021, the current national rate is suboptimal at 58.5% [13].

According to the National Immunization Survey-Teen (NIS-Teen) data from 2022, 76.0% of adolescents aged 13–17 have received at least one HPV vaccine dose with 62.6% having completed the series [14]. Comparatively, other adolescent vaccines such as Tdap and meningococcal are closer to 90% for receiving one dose. [14] Compared to the national percentage from the NIS-Teen 2022, Georgia’s HPV vaccine initiation and up-to-date rates among adolescents aged 13–17 are 70.8% and 61.5%, respectively [15]. Adolescents residing in rural areas compared to urban areas have lower initiation (68% versus 77.8%) and up-to-date (49.2% versus 60.4%) HPV vaccination rates from NIS-Teen 2020 [16]. Similarly, in the District of Albany (rural GA), only 47.9% adolescents aged 13–17 were up-to-date on their HPV vaccinations, which is 13% lower than the rest of the state, provided by Georgia Registry of Immunization Transactions and Services (GRITS) [17]. Therefore, this shows a gap in HPV vaccine uptake in rural communities and understanding the reasons behind low vaccine rates is crucial to increasing vaccination efforts.

Research has examined facilitators and barriers at the patient- (adolescent & parent), provider-, and practice-levels. The facilitators at both the patient- and provider-levels are patient’s trust in the provider, knowledge of the vaccine, and self-efficacy in one’s own ability to discuss the vaccine [18, 19]. For practice-level, the facilitators are the availability of the vaccine, scheduling future vaccine appointments, and prioritizing the vaccine [18]. The barriers at the patient- and provider-levels are the lack of knowledge and self-efficacy discussing the vaccine, concerns about safety and adverse effects, and not receiving provider recommendation for HPV vaccine [18, 20]. The barriers for practice-level are lack of access to vaccine provider, clinic logistics, and reminder system. [19] Few studies have explored facilitators and barriers of receiving the HPV vaccine intersecting at multiple levels of the socio-ecological model (SEM), and even fewer have been conducted in rural southwest Georgia [18, 21–24].

This qualitative study aimed to identify socio-ecological determinants influencing HPV vaccination uptake among parents, young adults, and public health professionals and providers in rural Georgia. We applied the P3 (patient-, provider-, practice-levels) Model to examine all three levels at the same time and how they impact each other, specifically around HPV vaccination [25]. At the patient level we assessed parents and young adults perceived susceptibility and severity. To assess all three levels we asked parents, young adults, providers and public health professionals about the facilitators and barriers of receiving the HPV vaccine series.

## Methods

We conducted a cross-sectional qualitative study to assess attitudes, knowledge, perceived severity and susceptibility, and reasons for HPV vaccination uptake (or lack of) among parents and young adults. In addition, interviews with healthcare providers and public health professionals were conducted to assess their knowledge, attitudes, practices, and the facilitators and barriers to HPV vaccination in rural communities. Between September 2020 to March 2022, a series of 40 interviews were virtually conducted with participants from southwest Georgia. At the beginning of the interview, the participant was told about the study, their role, risks and benefits of the study, and consented to participate. After consent was given, the interview was recorded on Zoom or an audio recorder. Emory staff (coordinators and students) were trained on the study, interview guide and conducted the interviews. The interviews were between 30–45 min and participants were compensated with a $25 electronic gift card. The study was deemed exempt by the Institutional Review Board at Emory University.

### Conceptual framework

**Fig. 1:**
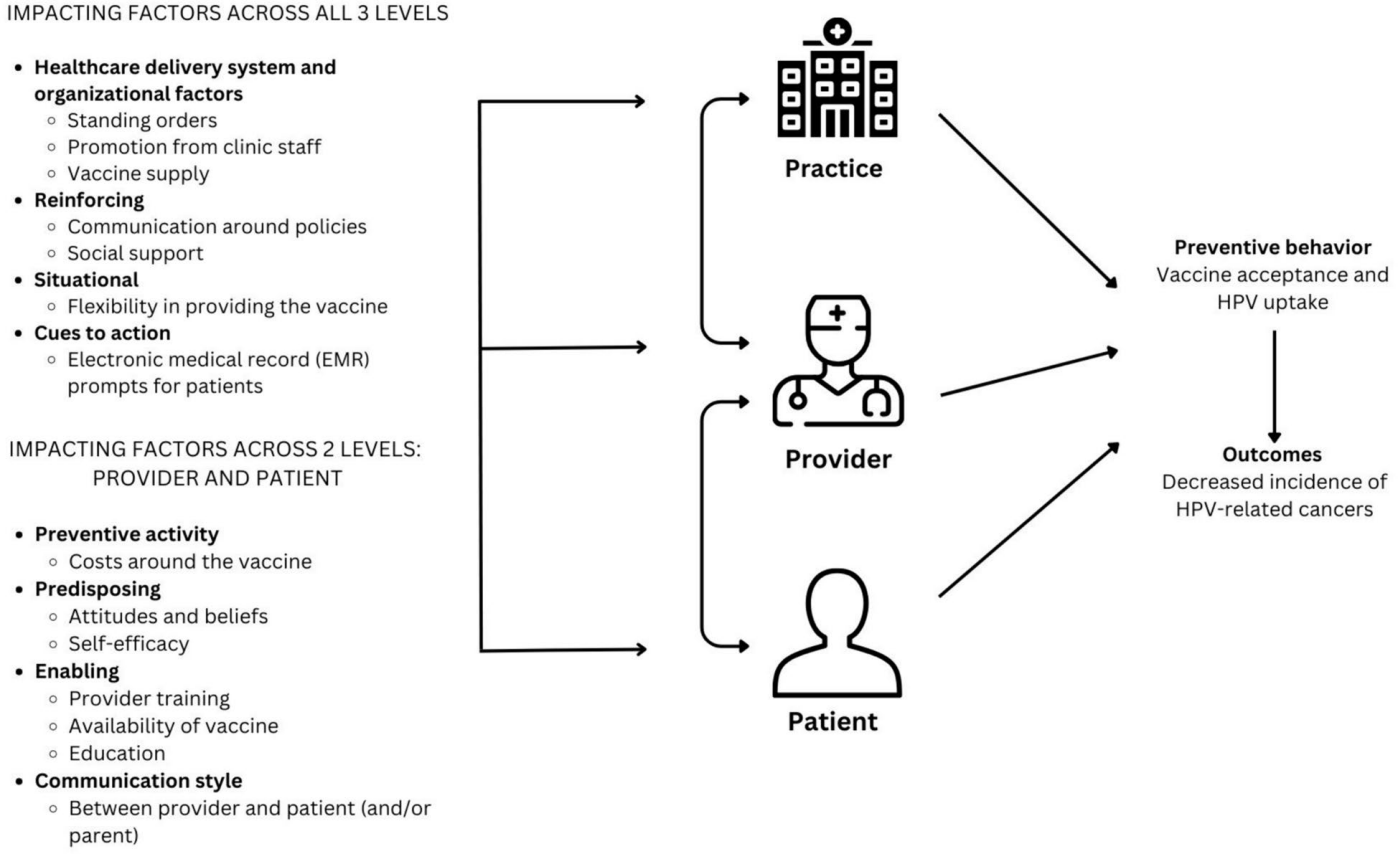
The HPV vaccine applied to the P3 (practice, provider, and patient level) model

This study was informed by the P3 Model and the SEM [25, 26]. The P3 Model is a unique approach since it encompasses not one but all three levels (patient, provider, and practice) of the clinic approach and integrates key components of health promotion and behavioral theoretical models (e.g., Health Belief Model, Theory of Planned Behavior, and ecological models (SEM) to impact health outcomes (Fig. 1) [25]. Since the P3 Model integrates multiple theories into the model, we utilized the model to guide our study and focused on targeting each of the levels in the model. The SEM describes the interplay of different levels of health factors that may influence the uptake of health behaviors at the individual, interpersonal, organizational (i.e., health systems), community, and policy levels [26]. From the SEM, we included questions beyond the P3 Model including community and policy-level factors that facilitate or hinder vaccine uptake. The frameworks applied to this study address limitations in existing rural health literature on HPV vaccination by considering healthcare system components beyond patient-level factors influencing parents’ and young adults’ vaccination decisions [25, 27].

#### Eligibility

This study included diverse participant categories from parents, young adults, providers and public health professionals. The parent of a child category was split into two groups: 1) vaccinated and 2) unvaccinated. The eligibility criteria for parents with a vaccinated child is a parent whose child received at least one dose of the HPV vaccine series and the child were between the ages 9–17. The eligibility for parents with an unvaccinated child is a parent whose child did not receive any doses of the HPV vaccine series and were between the ages 9–17. To be eligible for the young adult category, the person had to be between the ages of 18–34. Providers and public health professionals had to be a person who worked in a clinical setting or public health department or public health organization. The eligibility criteria of the interview sample are in Table 1.

**Table 1:** Interview sample and eligibility criteria

Interview Group	Sample Number	Eligibility Criteria
Parents with a vaccinated child	10	• A parent of a child aged 9–17
		• Has a child who has completed at least the first dose of the HPV vaccine series, if not all doses
Parents with an unvaccinated child	10	• A parent of a child aged 9–17
		• Has a child who did not receive any doses of the HPV vaccine series
Young adults	10	• A young adult aged 18–34
PCPs or providers at clinics or health department or public health staff	10	• Physicians, nurses, or other providers who work in a clinical setting or a person in a health department or public health organization

#### Recruitment

We used snowball and convenience sampling methods for participant recruitment and recruited only from southwest Georgia, which consists of 33 counties. Participants (e.g., parents and young adults) were recruited from the Emory Prevention Research Center (EPRC) Community Advisory Board (CAB), Facebook advertisements, and emails. The CAB is comprised of community members and leaders, health providers or staff from health systems, the public health district, businesses, and non-profits in southwest Georgia. This CAB has been in existence for over 20 years and members typically commit for two–three years. The Facebook advertisements were posted on the EPRC Facebook page targeting parents and young adults who live in southwest Georgia. Some parents, young adults, and providers were recruited from elementary schools and universities by receiving cold emails from the study team. In the email, eligibility and demographic questions were asked such as the age of the child/young adult, has the child/young adult received the HPV vaccine series, and if not, whether there are any plans for them to receive the HPV vaccine series in the future. The last two questions were about county of residence and race. These two questions were asked to make sure the study team captured a diverse sample. Public health professionals were recruited from non-profits and health agencies through word of mouth, fliers, and emails. To ensure saturation was met we had at least 10 participants for each category.

#### Interview guide development

The qualitative study had three interview guides for different audiences (parents, young adults, and providers/public health professionals). The interview guides were informed by the P3 Model and the socio-ecological model [25, 26]. The questions revolved around six topical categories, including: 1) knowledge, 2) facilitators to receipt of HPV vaccine, 3) barriers to receipt of HPV vaccine, 4) healthcare delivery factors, 5) community and resources, and 6) demographics. In addition, in the parent and young adult interview guides we asked about preventive care and interaction with providers around HPV vaccination. For providers, we also asked about promotional methods for the vaccine, the use of the Vaccine for Children’s program, staffing and supply issues, and if they have strategies or received training on how to talk to patients and parents about the vaccine (Table 2). Across all categories, we assessed participant demographics by asking demographic questions at the end of the interview before concluding the recording. The demographic questions included age, gender, race, and ethnicity (whether they are of Hispanic/Spanish descent). In addition, for providers and public health professionals, we inquired about their title and discipline, the organization they work for, and how long they have worked there. The interview guides and methods were reviewed by the study team and a working subgroup consisting of researchers from the EPRC and the EPRC CAB. The CAB members who participated are a healthcare provider, an infectious disease epidemiologist, and a health district deputy director. These CAB members, EPRC researchers, and our Emory team met three times before the data collection to guide the instrument development, recruitment methods, and data analysis plans. The results also were shared with them through several CAB meetings.

**Table 2:** Interview guide topics

Topical Domain	Example of questions
Knowledge	*What can you tell me about the HPV vaccine?*
	*Are you aware of cancers that are linked to HPV vaccine?*
Attitudes	*How well do you think the HPV vaccine works?*
	*How would you describe the HPV vaccine?*
Facilitators to Receipt of the Vaccine	*What education does your doctor provide about the HPV vaccination? Can you tell me about each of them?*
	*What materials have you seen or heard about the HPV vaccine in your community?*
Barriers to Receipt of the Vaccine	*What do you think gets in the way of children or teens getting vaccinated?*
	*What are barriers related to parents or teens in general?* Individual: Knowledge, costs, attitudes/thoughts about vaccine
	*What are barriers at providers’ offices or clinics?*
Healthcare delivery factors	*What education does your doctor provide about the HPV vaccine?*
Community and resources	*What materials have you seen or heard about the HPV vaccine in your community*
Demographics	Gender, age, insurance status, employment, education, # of children, children been vaccinated (self-report)
	Healthcare Provider/staff: title, type of organization, # of years at that organization

#### Analysis

All the interviews except for two were recorded on Zoom. The two interviews not recorded on Zoom were recorded on an audio recorder since the interviews were conducted over the phone. The interviews were then transcribed by a professional transcription service. We applied a systematic method for thematic data analysis including iterative codebook development with deductive codes from the interview guide, first-round coding, secondary coding, refinement of the codebook, consensus, final analysis, and matrices of themes [28]. A codebook with definitions was developed using a deductive coding approach from the three interview guides (parent, young adult, and providers/public health professionals) and the P3 Model and inductive codes. All transcripts were uploaded to MAXQDA for analysis [29]. Two trained researchers coded each transcript with the secondary coder reviewing coding from the primary coder. If there were discrepancies, then the coders would meet to discuss and come to an agreement and add new codes to the codebook when needed [28]. Emerging themes were identified for barriers and facilitators across each of the levels and finalized until saturation was reached [30]. Themes were sorted by facilitators and barriers and then broken down further by each of the levels in the P3 Model and socio-ecological levels (community and policy) in matrices with stronger themes ranked first.

## Results

We had 10 young adults, 20 parents, and 10 providers and public health professionals (health system participants) participating in the qualitative study. The young adults were 80% female and 20% male, 60% Black, and 40% White, 90% non-Hispanic and 10% Hispanic. The parents were 95% female and 5% male, 60% Black, 35% White, and 5% did not specify their race. Adolescents of the parents were 53% female and 47% male, 42% were ages 9–12, 48% were ages 13–17 and 10% were 18 and over. Health system participants were 90% female, 10% male, 60% Black and 30% White and 10% not specified. More than half of all participants and providers identified as African American (60%), about a third identified as White (35%), and 5% of participants did not specify their race. Additional demographics of the sample are displayed in Tables 3. and 4. The 40 participants reside in 11 out of the 33 counties in southwest Georgia and the top three counties are: Dougherty (52.5%), Lee (15%), and Colquitt (10%) seen in Supplemental Table 1.

**Table 3.** Demographics of participants

**Variable**		**Participant Demographics**							
		All Participants (*N* = 30)		Young Adults (*N* = 10)		Parents of Non-Vaccinated Children (*N* = 10)		Parents Of Vaccinated Children (*N* = 10)	
		**N**	**%**	**N**	**%**	**N**	**%**	**N**	**%**
**Age**	18–34	12	40%	10	100%	1	10%	1	10%
	35–44	9	30%	-	-	5	50%	4	40%
	45–54	7	23%	-	-	3	30%	4	40%
	55–64	2	7%	-	-	1	10%	1	10%
**Gender**	Female	27	90%	8	80%	9	90%	10	100%
	Male	3	10%	2	20%	1	10%	0	0%
**Race**	Black	18	60%	6	60%	5	50%	7	70%
	White	11	37%	4	40%	4	40%	3	30%
	Not Specified	1	3%	0	0%	1	10%	0	0%
**Ethnicity**	Non-Hispanic	28	93%	9	90%	10	100%	9	90%
	Hispanic	2	7%	1	10%	0	0%	1	10%
**Highest Level of Education**	Associates	4	13%	3	30%	1	10%	0	0%
	Some College	7	23%	0	0%	2	20%	5	50%
	Bachelors	12	40%	6	60%	3	30%	3	30%
	Masters	7	23%	1	10%	4	40%	2	20%
**Employment Status**	Full-Time	14	47%	3	30%	5	50%	6	60%
	Part-Time	4	13%	2	20%	1	10%	1	10%
	Unemployed	3	10%	1	10%	2	20%	0	0%
	Student	4	13%	3	30%	0	0%	1	10%
	Retired	1	3%	0	0%	0	0%	1	10%
	Self-Employed	3	10%	1	10%	2	20%	0	0%
	Unable to Work/Disabled	1	3%	0	0%	0	0%	1	10%
**Insurance Status**	Insured	28	93%	10	100%	9	32%	9	32%
	Uninsured	2	7%	0	0%	1	10%	1	10%
**Marital Status**	Single	16	53%	8	80%	3	30%	5	50%
	Married	9	30%	2	20%	4	40%	3	30%
	Divorced/Widowed	5	17%	-	0%	3	30%	2	20%
**HPV Vaccination Status**	Started Series	11	44%	5	56%	0	0%	6	38%
	Completed Series	14	56%	4	44%	0	0%	10	62%
**Number of Children per Participant**	1 child	10	50%	-	-	6	60%	4	40%
	2 children	9	45%	-	-	3	30%	6	60%
	3 children	1	5%	-	-	1	10%	-	-
**Ages of Children**	9 to 12	13	42%	-	-	8	53%	5	31%
	13 to 17	15	48%	-	-	6	40%	9	56%
	18 +	3	10%	-	-	1	7%	2	13%
**Gender of Children**	Male	15	47%	-	-	7	47%	8	47%
	Female	17	53%	-	-	8	53%	9	53%

**Table 4:** Demographics of providers

Variable		Provider Demographics	
		**Health Systems (*N* = 10)**	
		**N**	**%**
**Age**	18–34	1	10%
	35–44	4	40%
	45–54	2	20%
	55–64	1	10%
	65 and above	0	0%
	Unspecified	1	10%
**Gender**	Female	9	90%
	Male	0	0%
	Not Specified	1	10%
**Race**	Black	6	60%
	White	3	30%
	Not Specified	1	10%
**Ethnicity**	Non-Hispanic	9	90%
	Hispanic	0	0%
	Not Specified	1	10%
**Discipline/Training**	Administrator/Director	2	20%
	Doctor/Physician	1	10%
	LPN	1	10%
	NP	1	10%
	PA	2	20%
	RN	1	10%
	Not Specified	2	20%
**Years at Organization**	Less than 5	3	30%
	5 to 10	1	10%
	More than 10	4	40%
	Not specified	2	20%
**Organization Type**	Public Health	2	20%
	Health System	2	20%
	Community-Based	3	30%
	Healthcare Organization	1	10%
	Not Specified	2	20%

### Facilitators

There were facilitators for receiving the HPV vaccine identified at each of the three levels in the P3 Model. Facilitators at the patient level were having existing knowledge of HPV and the HPV vaccine, knowing the vaccine is safe, having knowledge on who can receive the vaccine and when, and having trusted individuals provide information about the HPV vaccine to their community. At the provider level, they were having efficacy of the vaccine, framing of the HPV vaccine to patients, and revisiting the HPV vaccination with hesitant parents. Facilitators at the practice level were immunization reminders, patient registries, the use of social media (e.g., educational videos), and other health clinics who support the vaccine. Immunization reminders was the most mentioned strategy mentioned across participants, both for the patients and providers to remind patients about the vaccine. See Table 5 for more facilitator quotes for each level.

**Table 5:** Facilitator quotes across levels

Facilitators			
**Themes**	**Level**	**Subtheme (if applicable)**	**Quotes**
Previous/existing knowledge of the HPV vaccine	Patient	Perception of the HPV vaccine	*“I’m going to say it’s [HPV vaccine] some insurance for your child’s behaviors and actions later on in life, you know? Lots of insurance.” (Participant 7, parent of vaccinated child)*
	Patient	Trusted people are the source of truth in the community	*“I think once they are educated, you know, by a trusted voice, you know, whether that’s their physician or, you know, pastor or somebody, whoever that trusted voice is for them, I think they’re more likely to be acceptable to that.” (Participant 39, health systems)*
Providers framing/approach on describing the HPV vaccine to parents	Provider	Providers framing the HPV vaccine as a preventative measure against other diseases	*“Just putting it out and putting the information out and let them stress that it is an STD just like any other STD. Of course, with repercussions in the future, and if you can prevent it, why not.” (Participant 6, parent of vaccinated child)*
Providers educating parents/patients on the HPV vaccine through various formats	Provider	N/A	*“And I try to re-educate if they didn’t, just because a lot of it is that they kind of don’t know what HPV is. They’ve heard of the vaccine. They understand that it’s a vaccine, but I don’t think they really know what HPV is and why they should be concerned about it.” (Participant 40, health systems)*
Health systems immunization reminders to patients/young adults	Practice	N/A	*“We have what’s called precall-recall, and so once a month we print out a list of our patients here that either they’re coming due for a set of immunizations they’ll be turning 11 in the next month. We’ll send out a letter that says your child will be due for immunizations on this day. We won't specifically say what immunization, but we’ll say they’re due for immunizations, can you please either call the clinic and make an appointment or come by. And then if they’re overdue we’ll send them a card that says your immunizations are past due. Please call our office and come by so that your – we can get your immunizations up to date.” (Participant 9, health systems)*
New ways to provide information about the HPV vaccine to parents/young adult	Provider	N/A	*“Public health [health department clinics] was probably our biggest champions of it, and that’s why we didn’t have to do an awful lot, particularly because we were focusing on lower income and uninsured patients. So, we had been able to get the community health centers to do as much as public health was doing, that would have made a huge difference…” (Participant 12, health systems)*
Implementing techniques for community engagement	Community	N/A	*“I’d say insight into the community, definitely, to get the word out. Because if you don’t have somebody from the community that also buys in, then they’re not going to participate, not going to show up” (Participant 25, health systems)*
Benefit of free vaccine programs	Policy	N/A	*“I think we have a free program with HPV…We get them (adult patients) to sign something and then we can get it for free for people who are uninsured” (Participant 23, health systems)*

#### Patient level

At the patient level, participants (primarily parents of vaccinated children and young adults both vaccinated and unvaccinated) consistently referenced having existing knowledge of HPV as a facilitator to increase uptake in administering the HPV vaccine. In describing the vaccine, participants referenced a basic understanding of which cancers it can prevent, and ages at which adolescents can receive the vaccine. Parents and young adults understood the safety of the vaccine, which assisted in having positive attitudes towards the vaccine. One parent described: *"I’m going to say it’s [HPV vaccine] some insurance for your child’s behaviors and actions later on in life, you know? Lots of insurance." (Participant 7, parent of vaccinated child).* For young adults, they learned about the vaccine on social media, and through school. Enlisting trustworthy individuals to connect community members with information within the community serves as another facilitator for increasing HPV vaccine uptake. A director of a non-profit alluded to this: *"I think once they are educated, you know, by a trusted voice, you know, whether that’s their physician or, you know, pastor or somebody, whoever that trusted voice is for them, I think they’re more likely to be acceptable to that*." *(Participant 39, health systems).*

#### Provider level

At the provider level, parents of vaccinated children highlighted the approach their children’s providers took when discussing the HPV vaccine with them. The providers framed the vaccine as a preventative measure against other diseases. Providers often spoke of the efficacy the vaccine has against contracting and spreading sexually transmitted infections (STIs), and how those STIs may have more serious ramifications later in life. A parent described this perspective: *"Just putting it out and putting the information out and let them stress that it is an STD just like any other STD. Of course, with repercussions in the future, and if you can prevent it, why not." (Participant 6, parent of vaccinated child)*.

In addition to framing the discussion, several education and messaging strategies were viewed as successful facilitators; these included patient visits at clinics, health departments, and utilizing community events to educate local community members. Some health system participants offered effective strategies such as revisiting the topic with hesitant parents and using information sheets to allow the parents to learn about the vaccine and its importance. One provider described their approach: *"And I try to re-educate if they didn’t, just because a lot of it is that they kind of don’t know what HPV is. They’ve heard of the vaccine. They understand that it’s a vaccine, but I don’t think they really know what HPV is and why they should be concerned about it." (Participant 40, health systems).* Parents of vaccinated children emphasized the use of brochures and pamphlets offered by providers as effective learning strategies. Some reflected on how this allowed for parents to take their time learning about the vaccine, and its benefits. Others viewed the brochure as a first step towards having a deeper conversation with the provider. Ultimately, parents thought brochures may bridge the gap for parents who do not know enough about the vaccine but want to learn more about it.

#### Practice level

At the practice level, immunization reminders sent to parents and young adults were seen as effective strategies by parents whose children were vaccinated and vaccinated young adults for patients to receive their HPV vaccine doses. Reminders included different formats depending on the health system, including phone calls and reminder cards. Health system participants also recognized different strategies to ensure patients returned for subsequent doses. These included the use of patient registries and highlighting those due for immunizations, as well as through the standardized Georgia Registry of Immunization Transactions and Services (GRITS), the statewide immunization information system. As one provider described their practice’s strategy:

*"We have what’s called precall-recall, and so once a month we print out a list of our patients here that either they’re coming due for a set of immunizations they’ll be turning 11 in the next month. We’ll send out a letter that says your child will be due for immunizations on this day. We won't specifically say what immunization, but we’ll say they’re due for immunizations…" (Participant 9, health systems)*.

#### Community and policy levels

At the community and policy levels, parents with vaccinated children and health system participants discussed techniques of using central and familiar locations like schools to engage in community outreach. Another one was to have champions within the community. One provider described: *“I’d say insight into the community, definitely, to get the word out. Because if you don’t have somebody from the community that also buys in, then they’re not going to participate, not going to show up” (Participant 25, health system nurse).* For policy, health system participants mentioned vaccine programs, explaining*: “I think we have a free program with HPV…We get them (adult patients) to sign something and then we can get it for free for people who are uninsured” (Participant 23, health system provider).*

### Barriers

The barriers for receiving the HPV vaccine at each of the three levels in the P3 Model were the lack of information and dialogue around the HPV vaccine. At the patient level, the main barriers were a dearth of education on HPV and the HPV vaccine, misinformation, and stigma as is relates to STIs and sexual intercourse. At the provider level, a deficiency exists in direct provider-patient communication, including instances where providers fail to inform and recommend the HPV vaccine to their patients. At the practice level, there are a lack of systematic reminders for patient immunizations reminders, limited information, time, staff, and resources committed to the HPV vaccine (Table 6).

**Table 6:** Barrier quotes across levels

Barriers			
**Themes**	**Level**	**Subtheme (if applicable)**	**Quotes**
Lack of education and knowledge	Patient	N/A	*“They’re (doctor) like, oh yeah, we now offer the HPV vaccine. Is it something you want to get? And my mom was like, eh, no, she doesn’t need that right now. And I was like, okay. I don’t really want a shot either, so it’s fine with me.” (Participant 17, unvaccinated young adult)*
	Patient	Misinformation surrounding the HPV vaccine	*“I think all of the conspiracy theories that are out there now, and it’s even worse since COVID, nobody trusts, or a lot of people don’t trust public health messages anymore.” (Participant 12, health systems)*
	Patient	Stigma surrounding HPV	*“Well, I think part of it is that since it is sexually transmitted, I think that a lot of parents don’t want to really delve into that thought that their kids are being sexually active or may be sexually active soon” (Participant 1, parent of vaccinated child)* “*I think the – I think stigma, because it is associated with sexual – a sexual nature. So, they kind of clam up like here in southwest Georgia, Bible belt, like it’s just kind of a – you know, you don’t speak of those things. Those are kind of taboo. Like everybody knows it’s occurring, but you don’t really want to I guess see your child doing – you know, doing things like that. So, I think it’s just the culture here.” (Participant 15, health systems)*
Lack of direct and consistent communication with the patient/parent	Provider	N/A	*“…they presented it, and asked did I want him to receive the vaccine, but at that time, I just had not had enough information on it personally, and with that, they did not give me any more information. And so, with that being said, you know, if my – if the doctor is not willing to provide more and give me more insight into it, any side effects, you know, statistics, and things of the sort, then you know, (laughs) yeah.” (Participant 2, parent of unvaccinated child)*
	Provider	Provider not advertising the HPV vaccine to patients	“*I think maybe lack of consistent recommendations. You know, they may get tied up in, you know, other bunch of check list of things that they’ve got to do and then may – it just may not be consistent throughout the flow…” (Participant 39 health systems)* *“I feel they should be more open and mention it in an exam. I do. I feel like they should. Not just have the poster up, like in the hallway. They still should mention it. The same way that they’re stressing the COVID vaccine, they should stress that vaccine in the same manner, I think.” (Participant 35, parent of unvaccinated child)*
Health systems immunization lack of reminders to patients/availability	Practice	N/A	*“Yeah. I think that like, for example, in my case, if there were an actual mailing that came to our house-…I would have seen it. I would have at least began a conversation with my husband about it, and he was the one responsible for taking him to the pediatrician and getting it handled.” (Participant 1, parent of vaccinated child)*
Limited time, information, staff, and resources	Practice	N/A	“*Time would be one I would see, because with a lot of the things that we’re having to do now, you don’t’ have as much time to do the education as you would like to, and sometimes when you’re talking about sex and HPV, if it’s on a one to one basis, it’s hard to establish a rapport in five, ten minutes and get all the information that you need to get to them and then allow them to ask questions” (Participant 25, health systems)*
Differences in private practices vs public health departments	Practice	N/A	*“If you’re more familiar with the doctor you have more trust, and you’re more likely to take their advice. When you go to one of the local clinics, the convenient care clinics, it’s not a guarantee you’re going to get the same doctor. So, you may not be as comfortable having a certain conversation with one doctor as you would with a doctor that you’re used to seeing on a regular basis” (Participant 13, Parent with a vaccinated child)* *“Private, is not private, and a lot of people may avoid going to the health department and would rather go to an outside pediatrician but don’t have the transportation to get there (Participant 6, Parent with a vaccinated child)*
Lack of transportation	Community	N/A	*“If I didn’t have a car, I probably wouldn’t even – I would barely go to the doctor if I had to use public transportation” (Participant 27, Parent with an unvaccinated child)*
Lack of community discourse on the HPV vaccine	Community	N/A	*“No, just that there is really not a lot of talks about it. I definitely think there needs to be more communication about it for sure” (Participant 17, unvaccinated young adult)*
Financial barriers	Policy	N/A	*“…some insurances don’t cover vaccines, so the parents have to end up paying out of pocket…” (Participant 15, health systems)*

#### Patient level

At the patient level, a persistent theme among parents of both vaccinated and unvaccinated children in our study focused on a dearth of knowledge among parents and their communities about the importance of vaccinating their children against HPV. They highlighted how it is not a common topic to be discussed among parents with their children. One young adult described their experience as a child, *“They’re (doctor) like, oh yeah, we now offer the HPV vaccine. Is it something you want to get? And my mom was like, eh, no, she doesn’t need that right now. And I was like, okay. I don’t really want a shot either, so it’s fine with me.” (Participant 17, unvaccinated young adult).* Not only is it not being discussed, but parents described not knowing where to go to find more information about the vaccine. Health system participants also discussed how parents often did not have the necessary knowledge about the vaccine to effectively make decisions on behalf of their children. Stemming from this lack of education is the impact that misinformation has surrounding the efficacy, safety, and utility of the HPV vaccine. Two non-vaccinated young adults address misinformation, one stated, “…*they’re [young adults] very hesitant about getting like even the COVID vaccine, just because, you know, they heard rumors, oh, it has this in it, it has that in it…*” another stated, *“They [young adults] look at social media and certain people may say this is what they do, this is what they don’t do, this is that. So, I think actually with social media and peer pressure that conveys a lot of the youth.”*

A director of a non-profit described, *“I think all of the conspiracy theories that are out there now, and it’s even worse since COVID, nobody trusts, or a lot of people don’t trust public health messages anymore.” (Participant 12, health systems).* In this context, the participant emphasizes the challenge of discussing the vaccine with parents and how a lack of trust in public health complicates messaging strategies.

Coupled with this misinformation was the resulting stigma of discussing HPV due to it being a STI. Vaccinated and unvaccinated young adults, both parents of vaccinated and unvaccinated children, and health system participants described how some parents may be reluctant to vaccinate their child, because they perceive it to indicate their child could be engaging in sex, or receiving the vaccine encourages the child to be sexually active. As one parent described, *“Well, I think part of it is that since it is sexually transmitted, I think that a lot of parents don’t want to really delve into that thought that their kids are being sexually active or may be sexually active soon” (Participant 1, parent of vaccinated child).* Particularly in southwest Georgia, sexual intercourse is stigmatized. As one provider described,

“*I think the – I think stigma, because it is associated with sexual – a sexual nature. So, they kind of clam up like here in southwest Georgia, Bible belt, like it’s just kind of a – you know, you don’t speak of those things. Those are kind of taboo. Like everybody knows it’s occurring, but you don’t really want to I guess see your child doing – you know, doing things like that. So, I think it’s just the culture here” (Participant 15, health system nurse).*

By attempting to discuss a vaccine to prevent STIs, health system participants believed this may contradict many who view teenage sexual health education as only relevant through abstinence.

#### Provider level

At the provider level, parents of unvaccinated children and young adults (both vaccinated and unvaccinated) alluded to the dearth of direct communication with providers about the vaccine and revisiting the topic with their patients. Specifically, some parents described how their child’s doctor did not educate them on the reasons for getting the vaccine. As one parent described their experience with a doctor as:

*"…they presented it, and asked did I want him to receive the vaccine, but at that time, I just had not had enough information on it personally, and with that, they did not give me any more information. And so, with that being said, you know, if my – if the doctor is not willing to provide more and give me more insight into it, any side effects, you know, statistics, and things of the sort, then you know, (laughs) yeah." (Participant 2, parent of unvaccinated child).*

This parent highlighted how they may have been convinced had the doctor provided more details about the reason for vaccinating their child. Another parent with an unvaccinated child described providers not revisiting the HPV vaccine with them at later visits if the parent initially said “no.” Aligned with the lack of direct communication, providers were not informing and recommending the HPV vaccine to patients. As a director of a non-profit stated, “*I think maybe lack of consistent recommendations. You know, they may get tied up in, you know, other bunch of check list of things that they’ve got to do and then may – it just may not be consistent throughout the flow…” (Participant 39, health systems).* A parent also felt the providers need to be speaking more about the HPV vaccine in the exam room. One parent described, *“I feel they should be more open and mention it in an exam. I do. I feel like they should. Not just have the poster up, like in the hallway. They still should mention it. The same way that they’re stressing the COVID vaccine, they should stress that vaccine in the same manner, I think.” (Participant 35, parent of unvaccinated child)*. Here, the parent wished the approach to HPV and the HPV vaccine was similar to the COVID-19 vaccine in order for them to understand its importance during their child’s visits.

#### Practice level

At the practice level, participants described lack of systematic reminders for patient immunizations, limited time, resources, and staff allocated per patient, and lack of education in the clinic or medical offices. A parent of each a vaccinated and unvaccinated child referenced not receiving vaccine reminders. One of the parents stated: *"Yeah. I think that like, for example, in my case, if there were an actual mailing that came to our house-…I would have seen it. I would have at least begun a conversation with my husband about it, and he was the one responsible for taking him to the pediatrician and getting it handled." (Participant 1, parent of vaccinated child)*. Although their child was vaccinated, the need for a mailed reminder would have facilitated discussions between the parents about vaccinating their child. Similarly, a young adult who received their first shot did not return for their second shot since they did not know when to return to the doctor’s office. Other barriers at the practice level include limited information, time, staff, and resources dedicated to the HPV vaccine. Both parents and health system participants mentioned time being a factor. One provider stated,

“*Time would be one I would see, because with a lot of the things that we’re having to do now, you don’t’ have as much time to do the education as you would like to, and sometimes when you’re talking about sex and HPV, if it’s on a one to one basis, it’s hard to establish a rapport in five, ten minutes and get all the information that you need to get to them and then allow them to ask questions” (Participant 25, health system nurse).*

As for the lack of resources, parents with a vaccinated child mentioned they have seen posters about measles, mumps, and rubella but not on the HPV vaccine and clinics not having enough of vaccines to distribute. A barrier widely mentioned across participants (parents, young adults unvaccinated, and health systems) were the differences between private practices and public health departments in rural communities. The differences between the two discussed were the patient-provider relationship and patient privacy differences. A parent explained:

*“If you’re more familiar with the doctor you have more trust, and you’re more likely to take their advice. When you go to one of the local clinics, the convenient care clinics, it’s not a guarantee you’re going to get the same doctor. So, you may not be as comfortable having a certain conversation with one doctor as you would with a doctor that you’re used to seeing on a regular basis” (Participant 13, parent with a vaccinated child).*

Another parent stated, *“Private, is not private, and a lot of people may avoid going to the health department and would rather go to an outside pediatrician but don’t have the transportation to get there (Participant 6, parent with a vaccinated child).* This parent explained health department layouts are openly structured and patients get called to a window to discuss their health information and people in the waiting room can hear those discussions, causing a lack of privacy for the patient, Similarly, a young adult unvaccinated also mentioned how privacy and courtesy of health professionals at certain clinics can be a barrier for patients. A lack of privacy is a concern at a patient level, while limited resources for transportation infrastructure affect the community at large.

### Community and policy levels

Several barriers at the community and policy level were mentioned by participants. At the community level the barriers include inadequate transportation, and lack of information within the community about HPV and the HPV vaccine and resources. A parent alluded to how important having a car is: *“If I didn’t have a car, I probably wouldn’t even – I would barely go to the doctor if I had to use public transportation” (Participant 27, parent with an unvaccinated child).* There is public transportation, but it takes more time and some unvaccinated young adults also stated how rural communities are spread out, which makes it challenging to travel to clinics that are out of their town and far away. A young adult described the lack of discussion around the vaccine in rural Georgia communities: *“No, just that there is really not a lot of talks about it. I definitely think there needs to be more communication about it for sure” (Participant 17, unvaccinated young adult).* At the policy level, the two main barriers participants mentioned were the financial barriers and lack of policies facilitating the uptake of the HPV vaccine. A provider described not being able to provide the vaccine to a minor without parental consent, *“…hey, we can’t give them to you, because you’re not 18. We can give you, you know, reproductive care, but we cannot give you any vaccine without your parents’ permission” (Participant 15, health system nurse).*

## Discussion

### Facilitators

Our study used the P3 Model framework and found common facilitators and barriers to receipt of the HPV vaccine in rural communities. Some of the facilitators we found were trusted individuals in the community, existing knowledge, and providers stating the vaccine is a cancer prevention tool. Parent participants from a study in Alabama reported that guidance from pediatricians or family physicians influenced their decision to vaccinate their children against HPV [31]. Another study in Montana noted parents may be more receptive to the HPV vaccine when it is discussed as a cancer prevention tool rather than an STI prevention tool [32]. A pivotal facilitator at the provider level in our study involved how providers phrase and frame the HPV vaccine to patients. Medical providers and public health stakeholders from a prior study in Montana identified a presumptive style of recommending the HPV vaccine. An announcement and conversation training HPV intervention for providers led to an increase in HPV vaccinations for adolescents ages 11–18 over those in a control group in North Carolina [33, 34]. This style included offering the HPV vaccine with other immunizations such as meningococcal, HPV, and Tdap together, which was successful [32, 34].

Additionally, our research revealed that immunization reminders were a key facilitator in improving HPV vaccination rates. A study in rural Alabama similarly reported that receiving appointment reminders via card, call, or text helped ensure all doses were received [35]. Similarly, healthcare provider participants from a study in Georgia highlighted the importance of scheduling subsequent HPV vaccine appointments before patients leave their first vaccination appointment and the use of reminder systems [18]. In addition to immunization reminders, health education material such as educational videos, and incorporating the use of social media were mentioned as strategies to engage people on the HPV vaccine by our participants. Our participants suggested that employing simplified strategies will better attract and engage the general population, especially those with lower health literacy. Previous research found that rural communities need increased access to education on HPV, the HPV vaccine, and sexual health [36].

In addition to examining facilitators within the P3 Model, we also examined community and policy-level factors. From our study, the community facilitators were trusted community key stakeholders and how they were instrumental in the development of interventions, [37] community and school education programs, [38] and county-wide social marketing campaigns [39]. Our participants mentioned having webinars and the use of school events and outreach is beneficial for increasing the uptake of the HPV vaccine. Future research could investigate interventions like technological reminders and capacity-building for rural healthcare systems to boost HPV vaccination rates. Additionally, future HPV vaccine promotion efforts could focus on community education and participation in campaigns such as the American Cancer Society’s Mission HPV Cancer Free [40]. At the policy level, some existing facilitators were the Vaccines for Children (VFC) program and private clinics instituting standing orders within their practice for the HPV vaccine [41]. The VFC was designed so children can receive vaccines regardless if the parent or guardian can afford the vaccines. Similarly, our study participants mentioned how beneficial vaccine programs are not only for children but for adults too.

### Barriers

Data from the 2010–2020 National Immunization Survey-Teen identified the following barriers to receiving the HPV vaccine: lack of knowledge, abstinence, safety concerns, and viewing the vaccine as unnecessary [42]. A lack of knowledge on HPV was reported as a prominent barrier among our rural participants, which has been observed across multiple rural-based studies [18, 19, 31, 35, 43]. Studies in rural Alabama found a lack of parental understanding about the HPV vaccine was a key barrier as reported by parents, pediatricians, and nurse participants [19, 35]. Provider participants from a quantitative study reported vaccinating adolescent females (13–17 years old) at higher rates compared to pre-adolescent females (9–12 years old) [44]. Barriers such as parental discomfort and potential adverse side effects in vaccinating their pre-adolescent child against HPV, especially if the child has underlying health conditions can influence the age group disparity in vaccine uptake [44, 45].

Another frequently discussed barrier in our study was stigma surrounding HPV as an STI and challenges in discussing sexual health, particularly given the conservative nature of southwest Georgia, located in the “Bible Belt” region. Previous research in the south (Georgia, Alabama, Kentucky, and North and South Carolina) found that parental perception of the HPV vaccination encouraging or permitting sexual activity discourages parents from having their child vaccinated against HPV [18, 31, 36, 43]. Prior research with healthcare providers from Georgia noted providers avoid discussing sex at all when recommending the HPV vaccine due to STI stigma [18].

Similarly, a lack of provider recommendations or discussion about HPV was a prominent barrier among our participants, consistent with previous literature [18, 35, 42]. According to providers in Georgia, low provider confidence in the HPV vaccine can pose a barrier to giving patients strong recommendations for the vaccine [18]. A national survey examining the quality of physician recommendation for HPV vaccination revealed that physicians in the sample often lacked consistency, urgency, and timeliness in their recommendation of the HPV vaccine [46]. Strategies such as education from other lay health professionals such as community health workers or navigators, training or mentoring of providers through technology, or partnering with other health organizations may be possible intervention strategies to explore [47]. Provider training on strong recommendations and presumptive communication has been effective in approaching this discussion about the HPV vaccine with parents and/or adolescents and effective in promoting vaccination [33, 48]. This type of training should be delivered to rural public health and healthcare providers to address these barriers [49].

Additionally, we found that a lack of patient reminders can hinder increases in HPV vaccination rates, which has been observed in prior studies. Reminder cards can easily be lost, so technology-based options, particularly email or text should be utilized based on individual patient preferences [43]. However, not all rural areas have the capacity to utilize text messaging based on limited cellar service [50]. Future efforts to increase HPV vaccination need to include relevant reminders for patients and/or caregivers. In addition to a lack of patient reminders, there is a lack of privacy within healthcare for patients. As shown in our study, patients commented on the lack of privacy in health departments and previous research highlights healthcare does not have appropriate privacy protections for patients [51]. Moreover, our participants noted that in rural areas, inadequate staffing and resources were also barriers to HPV vaccine uptake. A study in rural North and South Carolina also found that provider shortages in rural areas result in fewer opportunities for parents and adolescents to learn about the HPV vaccine [36].

From previous research, barriers at the community level consist of a lack of transportation and how it negatively affects people getting to their appointments to receive medical care [52, 53]. Participants in our study mentioned how a lack of transportation is a challenge, especially when one does not own a car or is unable to drive. Future research can explore methods to increase vaccinations outside of clinical settings including community settings or in pharmacies, as recommended by the President’s Cancer Panel report [54].

Similarly, a lack of policies can impact the uptake of the HPV vaccine. For example, the HPV vaccine is not routinely mandated for school entry at the state level, unlike other vaccines such as Tdap. While all states and the District of Columbia have middle school entry requirements for Tdap vaccination, only four U.S. jurisdictions (Rhode Island, Virginia, Puerto Rico, and the District of Columbia) currently have HPV vaccination requirements [41, 55]. Georgia did propose a bill in 2019 to allow adolescents younger than 16 to consent to vaccinations without parental consent, however, the bill did not pass [56, 57]. Due to this, minors will need parental approval to receive the vaccine and our participants explained the difficulty of this. Other policy barriers are financial vaccine burdens on health systems that administer the vaccines and lack of reimbursement from insurance companies [58]. Future implementation and evaluation of HPV policies (e.g., school or policy requirements) could assess policy solutions and impacts on HPV vaccine uptake.

### Strengthens and limitations

The strengths of this study include interviewing three categories of community stakeholders and receiving their insights on the facilitators and barriers of the HPV vaccine in rural Georgia. In addition, for parents and young adults, we received perspectives from those who received and did not receive the HPV vaccine. Using the P3 Model in our study and the subsequent findings, allowed for consideration of multi-level interventions for increasing HPV vaccination. Research has shown numerous programs that promote HPV vaccination operate at a single level [59]. Mostly focusing on either the patient or provider levels and a lack of focus on including facilitators and barriers at the practice level [60–62]. Public health professionals and providers can learn from these facilitators and barriers to test strategies at different levels to increase rural HPV vaccination rates. However, our study has some limitations. The study findings may not be generalizable outside of rural southwest Georgia to other states or regions. There were also delays in recruitment because of participants’ limited time due to the impact of the COVID-19 pandemic. We had to pause recruiting providers because the community asked us to due to the demands of the pandemic and we focused our efforts on recruiting parents and young adults for the study. Participants may have offered socially desirable responses during the interview regarding the HPV vaccine. Finally, although we used several methods to increase the reliability of the qualitative data analyses such as using verbatim transcripts, a structured, iterative codebook and training of coders, and two research team members coding each interview, there may be research biases in the analyses and interpretation of our data.

## Conclusion

Identifying multi-level facilitators and barriers influencing HPV vaccination is necessary for increasing vaccine uptake, particularly in rural areas where vaccine coverage is disproportionately low. We found some key barriers at all three levels of the P3 model including misinformation, lack of knowledge, provider-patient communication, provider recommendation, lack of systematic reminders, and limited time and resources. These barriers highlight the need for future research to explore the effectiveness of the following strategies in rural communities: HPV vaccine education in rural communities through public health providers, provider training on strong recommendations, and technological health systems activities such as patient reminders.

### Supplementary Information

Supplementary Material 1.

## Data Availability

The data supports the findings of this study are available in tables and supplementary materials of this article. We can share general data matrices or summaries of the qualitative data if there are requested. Since this is a qualitative data and there is sensitive information about the vaccine and perhaps health systems, we will not share the actual transcripts. Contact Cam Escoffery at cescoff@emory.edu about data availability.

## Abbreviations

CAB: Community advisory board
HPV: Human papillomavirus
P3 Model: Patient-provider-practice-level Model
SEM: Socio-ecological Model
STI: Sexually transmitted infections
