# Supplementary material for: Facilitators and barriers of HPV vaccination: a qualitative study in rural Georgia
Source: BMC Cancer. 2024 May 15;24:592. doi: 10.1186/s12885-024-12351-1 (PMC11094994; doi:10.1186/s12885-024-12351-1)
Supplement: Supplementary file 1 — Supplementary Material 1. [file 12885_2024_12351_MOESM1_ESM.docx]

| **County** | Clay | Colquitt | Dougherty | Lee | Mitchell | Terrell | Tift | Turner | Thomas | Randolph | Seminole |
| --- | --- | --- | --- | --- | --- | --- | --- | --- | --- | --- | --- |
| **Total n, %** | 1(2.5%) | 4(10%) | 21(52.5%) | 6(15%) | 1(2.5%) | 1(2.5%) | 1(2.5%) | 2(5%) | 1(2.5%) | 1(2.5%) | 1(2.5%) |

Supplemental Table 1. County Representation
